# Observations on Medical Reform

**Published:** 1808-01

**Authors:** 


### *To the Editors of the Medical and Phyſical Journal.*

Gentlemen,

It has been justly remarked, and the opinion has of late been actively disseminated and widely promulgated, that the present condition of medical education and practice in the British dominions admits of much improvement. A subject so interesting to the feelings of every medical man, has naturally excited a considerable sensation among the profession, who seem very generally to concur in the necessity for reformation, although they certainly differ very widely as to the means for accomplishing this desirable reform. On a question so intricate, and involving such various, complex, and extended considerations, it was naturally to be expected that much diversity of opinion should arise. This indeed seems inevitable, even admitting that the individuals, who favour the world with their sentiments, are uniformly influenced by pure and disinterested motives. I fear, however, this is more than we can concede; and few I think will be hardy enough to deny, that amid such communications, we have to expect much declamation resulting from heated imaginations, and imperfect acquaintance with the subject; much misconception, the offspring of prejudice, of confined views, and limited experience; much wilful misrepresentation, flowing from illiberality of sentiment, and an exclusive attachment to self interest, with very little of truth or just reasoning, in proportion to the great mass of observations which this question is sure to elicit. Yet, should not any of these communications be repressed or discouraged by those who have taken a leading part in this inquiry; neither should they be received with harshness or ridicule. These advocates for reform should be satisfied with directing inquiry to the proper object, without being themselves under the influence of any preconceived system of reform, either in dictating their inquiries, or in judging of the communications these may give rise to. It is hardly to be expected, in an inquiry immediately addressed to both the social and self-interested feelings of the profession, that every writer shall evince perfect calmness of temper, unelated by the favorite ſpeculations of his own fancy, or unruffled by the real or supposed delinquency of some fellow labourer, whose narrow and illiberal schemes may call forth his reprehension.

sion. It is from such jarring elements, as the philosophic mind will readily perceive, that order will ultimately arise; it is from such a chaos of heterogeneous materials, that the real and upright friends of rational reform will finally be enabled to erect a fabric, durable in its structure, and appropriate to the ends for which it shall be designed. No evil can possibly arise from the most futile or the most rancorous effusions, so long as the suggestions of either are sure to be submited to the ordeal of public discussion, previous to their incorporation into our legal code; while from every communication, however trifling, may be extracted some hints conducive to the general issue, either as militating against or confirming those opinions which may be previously entertained.

These simple and obvious considerations I woud humbly submit to all those whose situations authorise them to sit in judgment on the several communications which have appeared, or which may hereafter issue from the press on the subject of medical reform. These I would also propose to the good sense of Dr. Harrison and his Committee, as not undeserving their attention, without meaning, however, to insinuate that their conduct has been such as to call for any remonstrance of this nature.

While such a line of conduct shall be adopted and adhered to, so long may the friends of medical science and liberal sentiment rest satisfied, that the question is undergoing that investigation which is best calculated to illustrate its real interests; and as such fair and unrestrained discussion must manifestly lead to the early detection of all errors, whether of ignorance or design, little apprehension need be entertained of any crude or sinister measures being precipitately imposed on the legislature, under the mask of liberal and enlightened reform. Much time must elapse, and much controversial discussion be endured and examined, ere any rational and effectual reform of the medical profession can be digested and arranged; and better is it by far to await patiently such a consummation of our purposes, than by an over zeal to correct existing errors and abuses, hazard not only the immediate interests of the profession, and the welfare of soeiety, but also the rendering a real and effective reformation both more uncertain and more remote.

The reality of such errors and abuses cannot be disputed, and yet this admission affords no plea for any undue haste or precipitation in the attempts to reform these. By many, (as must always be the case in all such exhibitions) they they have been magnified beyond all truth. But admitting them to be as erroneous as even the most zealous reformists would wish to depict them, still this consideration so far from warranting a hasty appeal to the legislatnre, should only render us the more cautious and circumspect in devising remedies adequate to meet the evils complained of.

It should be borne in mind too, that in undertakings of this nature, the measure of success bears no proportion to the energy with which the ultimate object is pursued, but that it is much rather dependant on the wisdom of our arrangements, aud the steadiness and deliberation with which we proceed to carry them into effect. So much opposition, however, has arisen to the views of the reforming society, and to the outline whieh they have furnished, and so irreconcileable thereto do several of our great chartered bodies seem to be, that it may appear scarcely required thus to enforce the necessity for deliberation, when means so much more powerful than any individual remonstrance could ever be, concur to render this a matter of certainty. True it is, that in the present aspect of affairs, there seems to exist little danger of precipitation. Nor was it merely to avert this that I entered on the present communication; I had it equally in view to point out to the advocates of reform, that such opposition was necessarily to be expected, and to reconcile them thereto, by demonstrating the advantages derivable from all such collusion of sentiment; to warn them against the effects of those passions which controversy, and, what they may deem vexatious opposition, may too probably excite; to exhort them not hastily to abandon the cause they have undertaken, but to pursue it with that patient spirit of investigation which can only lead to ultimate perfection. In order to render their prospect of success greater, and to increase the number of liberal supporters, I would strenuously advise them to expand their views, and to take a more elevated station, so as to do away that reproach to which heretofore they have rendered themselves but too liable, namely, of being associated for the contemptible purpose of forming "*a pitiful plan* for the *good of the trade*." They cannot surely be ignorant, that in all institutions arising out of the wants of society (of which the medical profession is assuredly one), the well-being of that society is the great and paramount consideration to which every other should unquestionably be rendered subservient; and that any reform of the profession which has not this fundamental truth for its basis, and which does not keep keep this stedfastly in view, must of necessity be incomplete, if not subversive of its avowed purpose and design. That such plans of reform should meet with strenuous opposition can never excite surprise; while it must afford matter for triumph rather than regret, when such are detected in their futile propositions, and insincere professions, and are consequently rejected with the contempt they merit. Mere good intentions can furnish no plea, in the present advanced state of political science, for extending unmerited indulgence to speculative propositions, marked only by inanity of design, and which affect to perform what they are utterly inadequate to accomplish. On the contrary, the good of mankind requires, that every error so advanced should be promptly investigated and unequivocally exposed. Nor will such exposure affect the authors of such propositions with aught but pleasure at the timely detection, and gratitude to the expositors, so long as the views of the former are liberal, and their avowed wishes sincere. As an evidence of the purity of their intentions, and the liberality of their views, let the present heads of the reforming association conduct their future inquiries in such manner as that the addresses they may circulate, shall bear no obvious affinity to those conclusions which their own private sentiments may make them desirous of establishing. Let their queries be so couched, and in such general terms, as that the informant shall be under no influence in returning his responses, save that of a strict regard to truth and integrity. Let them not first prejudge the question, and then institute those inquiries which should ever precede judgment, and on whose result this should ever be founded. Above all, let them receive with equal candour, and give equal publicity to all communications which may reach them, whether these may accord with their general and received opinions, or militate against them. Let them regard themselves, not as a tribunal competent to sit in judgment, and at whose bar the whole medical profession, whether regular or irregular, is bound to plead, but as a mere committee of inquiry, associated for the purpose either of confirming or disproving (indifferently as the event may determine) the reality of abuses alleged to exist. As such, even the self-constituted, and possibly too coming forward under some suspicious appearances, yet the more enlightened and valuable members of the profession willingly assist their inquiries with all necessary information.

So also it is to be hoped would the proficients in political science science afford them just views respecting those ordinances by which this complicated profession ought to be regulated. When by such patient investigation, and by perseverance in such efforts, a full body of information shall have been accumulated; and when, by the collision of opposing doctrines and opinions, all early prejudices and unfounded impressions shall have been done away, and truth shall appear unobscured by the darkness of falsehood and error; then will the period arrive for digesting the outline of either a partial or general reform in the medical profession, according as circumstances may seem to require; then will it be meet and right to make an appeal to the legislature to interpose their authority, and to give efficacy to measures, which can no longer be deemed of a doubtful or suspected nature.

This cautious procedure will, doubtless, but ill accord with the ardour of those, who, viewing as in a magnifying reflector, the dark detail of enormities said to be daily practising in the profession of medicine, or under the sanction of its name, and which, probably, appear the more amplified from being now, for the first time, perhaps, stedfastly regarded, feel all their humanity aroused against the hydra of quackery; and who, consequently, from the best motives, are strenuous in urging on its immediate destruction. But let such enthusiasts beware, lest, in attempting partially to decapitate this monster, they but multiply its baleful powers; and let them yield conviction to that truth, which human experience has so amply confirmed, and which is so beautifully illustrated by the mythologic fable, from whence the foregoing metaphor is derived ; namely, that mere force or power (whether physical or political), undirected by wisdom, can never succeed in repressing the encroachments of evil, or in compassing its destruction. This conviction should render the reformists cautious of trusting, too implicitly, to the efficacy of legislative restrictions and penalties, as opposing an adequate resistance to the irregular practice of medicine; and will have the effect, I trust, of inducing them to digest and mature their plans sufficiently, before the strong arm of the legislature is employed to enforce these.

Thus far have I ventured to offer my sentiments, as to the views by which the reforming association, and all who undertake a similar province, should be guided; and also as to the mode in which the necessary investigations ought to be conducted. The opinions I entertain respecting the objects of that inquiry, I have elsewhere fully detailed; with a degree of haste and precipitancy, indeed, which can only be justified on those principles of investigation, which it has been one of the objects of this paper to support.

I meant them not as perfect propositions, incapable of amendment; neither did I dictatorially advance them, as superseding the necessity for further investigation. I merely brought before the public the sentiments of one individual, which being unsupported by any name or sanction, must stand or fall by their own merits. I own I was not over anxious to reconsider my general theories, or to harmonise the language in which they were conveyed. Conscious that my errors, if of magnitude, must soon be detected, I allowed no false apprehensions respecting them to make me withhold, for a moment, those truths, which I flatter myself I have not been altogether unsuccessful in developing. The general principles on which I had reasoned I knew the justness of, and did not distrust; and with these for my guides, I was the less afraid of falling into material errors. Nor do I, on revision, perceive that I have committed many of importance.

With some solicitude, I confess, have I awaited the manifestation of those errors, by the several public censors who have assumed the task of passing judgment on all such; but hitherto my wishes, in this respect, have not been gratified.

For the brevity of the analysis which your Journal afforded, the nature of your publication, which is but in part critical, is an abundant excuse. But from that review, which is more exclusively devoted to medical critique, and which has taken so active a part in detailing the proceedings which have been entered into by the reforming association, I did expect something of a fair and comprehensive statement of my general principles, with an analysis sufficiently copious and general, to illustrate these. In all of which, however, I was doomed to meet with disappointment, being favoured only with a mutilated statement of partial details, unconnected with any of the principles on which they rested; together with some egregious and uncalled for misrepresentations of certain insignificant introductory explanations, which had arisen out of local circumstances, and which had a local reference only. In fine, I was given to understand, that as my views were more extended, and not very coincident with those of Dr. Harrison, I was to be regarded not only as a reformist but a rea revolutionist. I had advocated the causes of surgery and midwifery, assigning to each what appeared to me its just and relative situation; consequently, I was connected with both of these branches of the profession, in practice at least.

I had given a decided preference to the Edinburgh school of physic, as approaching nearest to perfection of any of the regular established schools in these countries; consequently, the University of Edinburgh must be my alma mater.

These instances of sagacity were, no doubt, great and notable; how much they were to the purpose, I leave to the discerning of my readers to discover. They should merely have excited my silent admiration, had I not perceived, in the whole critique, a marked prejudice against my observations, manifestly arising from a decided predilection for the plans of Dr. Harrison. Even this bias should have passed unnoticed, but for certain conjectures and misrepresentations in which the Reviewer had indulged himself; and which had a direct and manifest tendency to extend his prejudices to the public, while no sufficient opportunity was afforded them for correcting such an impression, by the exercise of their own judgments. In consequence, therefore, of my apprehensions, lest a cause, in which I felt considerable interest, should suffer from these misrepresentations being acquiesced in, which, after all, might not have been wilful, but owing, perhaps, solely to the necessary hurry of periodical composition, I ventured to forward to the Reviewer a few lines of explanation and remonstrance; requesting, either that he would insert them as a counterpoise to those deviations from sound criticism, which I had remarked, or that he would himself correct the errors he had fallen into, aided by the explanations I had afforded him. This not unreasonable request, however, produced an effect I looked not for; it seemed to irritate the Reviewer, as bringing in question his infallibility; and was only noticed by a brief reference to one only of those misrepresentations I had complained of. In reverting to this, he attempts even a justification of his first judgment, when overlooking the possibility of medical reformers, worthy of notice, existing out of London, or beyond the pale of Dr. Harrison's sect, he supposed certain expressions of mine as alluding to the association in Soho-square, while I had only in mind a very different association, one much nearer home, and from whose *secret, insidious, and undermining policy* (words

 surely surely of plain import, but which the Reviewer was pleased to denominate a rhapsody), I had much greater reason to apprehend danger to the profession in that country, which was to me more immediately an object of interest.

In proof of his first application being the most probable, he again produces the words themselves; and yet he had before shewn, clearly and satisfactorily, that in the sense in which he had taken them, they were utterly inapplicable! He finally makes an appeal to all readers, on his own side the water; and supposing that they must all commit the same oversight, which he himself had been guilty of, he feels satisfied they will all interpret, as he has done, this unhappy passage, so unimportant to the general objects of the Essay in question, and so truly undeserving the extraordinary notice it has been honoured with.

To this gentleman I bear no enmity; and if my language to him was intemperate, as he asserts, I here declare that I was unconscious of its being so; and that to give the least offence, was far from my intention.

It may now be asked, to what end is all this elucidation of so insignificant a subject?—to which I answer, that it was in contemplating the conduct of this Reviewer, and in observing the consequences, which had so clearly flowed from his having yielded up his mind to the influence of prejudice and of hasty impressions, that I was lead more immediately to consider the necessity for keeping the mind disengaged from all preconceptions, pending the discussion of so important a subject.

It was in following out the train of reasoning, thus commenced, that I was lead into making the foregoing reflections and observations; and it was, in fine, to illustrate the whole subject more clearly, and to render my cautions and suggestions more intelligible and more obvious in their application, that I have, in the last instance, detailed the communications which I held with the London Reviewer; which, but for this motive, which I trust will justify the insertion, would otherwise, in such a place, have been highly impertinent and irrelative. As it is, I hope I may expect forgiveness, both from the reviewer and the public; and that the obvious propriety of those hints, which I have, in the foregoing pages, ventured to propose, will procure them the attention they may deserve, however the writer of them may, in other respects, be adjudged as meriting reprehension.

The sanction of a name being, in the present instance, utterly utterly unnecessary, and not having the vanity to conceive that mine could give confirmation, or extend a fostering protection to any opinions I may have here, or elsewhere, offered on the subject of Medical Reform, I shall continue, therefore, to employ my former signature, and to subscribe myself, Gentlemen,

